# Effects of a Rehabilitation Programme Using a Nasal Inspiratory Restriction Device in COPD

**DOI:** 10.3390/ijerph18084207

**Published:** 2021-04-15

**Authors:** Jose L. Gonzalez-Montesinos, Jorge R. Fernandez-Santos, Carmen Vaz-Pardal, Jesus G. Ponce-Gonzalez, Alberto Marin-Galindo, Aurelio Arnedillo

**Affiliations:** 1 Department of Physical Education, Faculty of Education Sciences, University of Cádiz, 11519 Puerto Real, Spain; jgmontesinos@uca.es; 2 Galeno Research Group, Department of Physical Education, Faculty of Education Science, University of Cádiz, 11519 Puerto Real, Spain; 3 Biomedical Research and Innovation Institute of Cádiz (INiBICA) Research Unit, Puerta del Mar University Hospital, University of Cádiz, 11009 Cádiz, Spain; jesusgustavo.ponce@uca.es (J.G.P.-G.); aurelioarnedillo@neumosur.net (A.A.); 4 Bahía Sur Andalusian Center for Sports Medicine, 11100 Cádiz, Spain; carmenvaz@hotmail.com; 5 MOVE-IT Research Group Department of Physical Education, Faculty of Education Science, University of Cádiz, 11519 Puerto Real, Spain; amaga7208@gmail.com; 6 Pneumology, Allergy and Thoracic Surgery Department, Puerta del Mar University Hospital, 11009 Cádiz, Spain

**Keywords:** chronic pulmonary obstructive pulmonary disease, inspiratory muscle training, physical exercise, cardiopulmonary exercise test

## Abstract

Chronic obstructive pulmonary disease (COPD) patients are characterised for presenting dyspnea, which reduces their physical capacity and tolerance to physical exercise. The aim of this study was to analyse the effects of adding a Feel-Breathe (FB) device for inspiratory muscle training (IMT) to an 8-week pulmonary rehabilitation programme. Twenty patients were randomised into three groups: breathing with FB (FBG), oronasal breathing without FB (ONBG) and control group (CG). FBG and ONBG carried out the same training programme with resistance, strength and respiratory exercises for 8 weeks. CG did not perform any pulmonary rehabilitation programme. Regarding intra group differences in the value obtained in the post-training test at the time when the maximum value in the pre-training test was obtained (Post_PRE_), FBG obtained lower values in oxygen consumption (VO_2_, mean = −435.6 mL/min, Bayes Factor (BF_10_) > 100), minute ventilation (VE, −8.5 L/min, BF_10_ = 25), respiratory rate (RR, −3.3 breaths/min, BF_10_ = 2), heart rate (HR, −13.7 beats/min, BF_10_ > 100) and carbon dioxide production (VCO_2_, −183.0 L/min, BF_10_ = 50), and a greater value in expiratory time (Tex, 0.22 s, BF_10_ = 12.5). At the maximum value recorded in the post-training test (Post_FINAL_), FBG showed higher values in the total time of the test (T_t_, 4.3 min, BF_10_ = 50) and respiratory exchange rate (RER, 0.05, BF_10_ = 1.3). Regarding inter group differences at Pre_POST_, FBG obtained a greater negative increment than ONBG in the ventilatory equivalent of CO_2_ (EqCO_2_, −3.8 L/min, BF_10_ = 1.1) and compared to CG in VE (−8.3 L/min, BF_10_ = 3.6), VCO_2_ (−215.9 L/min, BF_10_ = 3.0), EqCO2 (−3.7 L/min, BF_10_ = 1.1) and HR (−12.9 beats/min, BF_10_ = 3.4). FBG also showed a greater Pre_POST_ positive increment in Tex (0.21 s, BF_10_ = 1.4) with respect to CG. At Pre_FINAL_, FBG presented a greater positive increment compared to CG in T_t_ (4.4 min, BF_10_ = 3.2) and negative in VE/VCO_2_ intercept (−4.7, BF_10_ = 1.1). The use of FB added to a pulmonary rehabilitation programme in COPD patients could improve tolerance in the incremental exercise test and energy efficiency. However, there is only a statically significant difference between FBG and ONBG in EqCO_2_. Therefore, more studies are necessary to reach a definitive conclusion about including FB in a pulmonary rehabilitation programme.

## 1. Introduction

Chronic obstructive pulmonary disease (COPD) is considered as one of the main causes of morbidity and mortality worldwide [1]. This disease is characterised for causing a chronic limitation of the air flow, which leads to dyspnea, mainly when making an effort, thus reducing physical capacity and worsening the quality of life [2]. Training with physical exercise is considered one of the pillars within pulmonary rehabilitation programmes in the treatment of COPD, since it is an effective way of delaying physical deterioration and the consequent functional limitation in COPD patients [1,3].

The standard prescription of physical exercise includes cardiorespiratory capacity training, through either walking or cycling, and muscle strength training, for both the upper and lower limbs [4]. Additionally, inspiratory muscle training (IMT) has proved to be an effective method to reduce dyspnea, increase physical capacity and improve the quality of life [5,6]. However, IMT is rarely included in pulmonary rehabilitation programmes, since it does not seem to provide an additive effect when used with other forms of training [6,7,8,9]. This result can be partly due to the fact that IMT is carried out separately and not concurrently with exercise, which has been shown to have an ergogenic effect on healthy people [10]. In fact, there are different devices for IMT in COPD patients, although the training is performed in a static position, usually with the individual sitting, thus without simultaneously activating the skeletal muscles involved in the exercise for both cardiorespiratory capacity and muscle strength training.

In a recent study, a ventilatory flow filtering and restricting device called FeelBreathe^®^ (FB) (University of Cádiz, Cádiz, Spain), which is placed under the nostrils, was designed to increase the resistance to the nasal air flow [11]. This device is made of hypoallergenic material and designed in different sizes and porosity. The larger one makes inspiration more difficult. Previous studies in COPD patients using FB have reported improvements in ventilatory efficiency and pattern, as well as increased inspiratory and expiratory time during exercise [12]. FB has also been reported to provide improvements in dyspnea, quality of life, exercise tolerance and inspiratory muscle strength after using it in an 8-week pulmonary rehabilitation programme [13].

Cardiopulmonary exercise testing has become a key tool to analyse the efficacy of pulmonary rehabilitation programmes in COPD patients, since it allows monitoring the metabolic and cardiorespiratory response with the aim of identifying patterns of functional deterioration and measuring exercise tolerance [14,15]. In fact, there are specific guidelines to obtain and interpret results in this test with COPD patients [16,17]. The limitation in this type of exercise in a COPD patient is related to an inefficient exchange of gases in the lungs, muscle deconditioning and dynamic pulmonary hyperinflation [15]. An effective training programme will produce adaptations related to these physiopathologies with the aim of improving the general physical performance.

Therefore, the aim of this study was to analyse the effects of using a FB device, in an 8-week pulmonary rehabilitation programme, on the exercise performance of COPD patients to tolerate exercise, measured through cardiopulmonary exercise testing. Our hypothesis is that the use of FB during training will have an additive effect, producing greater adaptations in the respiratory muscles and, thus, an even greater increase of the physical capacity compared to the training without FB.

## 2. Materials and Methods

### 2.1. Sample Size Calculation

Sample size calculations were performed for intra-group and inter-group differences using G*Power software version 3.1 (University of Düsseldorf, Düsseldorf, Germany). According to the results of the F test, a sample of 30 participants is needed to obtain a significance difference between measures obtained intra-group (statistical test: analysis of variance (ANOVA), repeated measures, within factors. Required input parameters: effect size = 0.25, level of significance α = 0.05, power β = 0.80, number of measurements = 3, correlation among repeated measures = 0.5). Regarding inter-group differences, a sample of 108 participants is needed to obtain a significance result (statistical test: ANOVA, repeated measures, between factors. Required input parameters: effect size = 0.25, level of significance α = 0.05, power β = 0.80, number of groups, number of measurements = 3, correlation among repeated measures = 0.5). 

### 2.2. Design

This study was designed as a clinical trial (NCT 01695265) in which the participants were assigned to the following three groups by stratified randomisation: physical training and IMT with FB (FBG); physical training without IMT (ONBG); and control group, i.e., no training (CG). FB has been authorized for use in the present study by the Spanish Agency for Medicine and Health Products (File 521/15/EC, AEMPS, Madrid, Spain). The randomisation process was performed using the randomizeR package for programming language R [18]. All patients, before initiating the rehabilitation programme, received a document with information about the study, and, after agreeing to participate in it, they signed the informed consent form. This clinical trial was approved by the Ethics and Research Committee of the University Hospital Puerta del Mar (Cádiz, Spain), and met the requirements of the declaration of Helsinki and the data protection law.

### 2.3. Participants

The inclusion criteria to participate in this study were as follows: patients diagnosed with COPD according to the GOLD (Global Initiative for Obstructive Lung Disease) criteria [2], between 35 and 70 years old, degree of dyspnea ≥2 in the modified dyspnea scale [19] and stable clinical condition for at least 2 months. On the other hand, the study excluded the patients with the following characteristics: any possibility of heart disease, neuromuscular or skeletal diseases that limited the patient’s physical performance, dyspnea at rest, the need for supplementary oxygen, CO_2_ retention or the use of any mechanical ventilation support.

**Figure 1:**
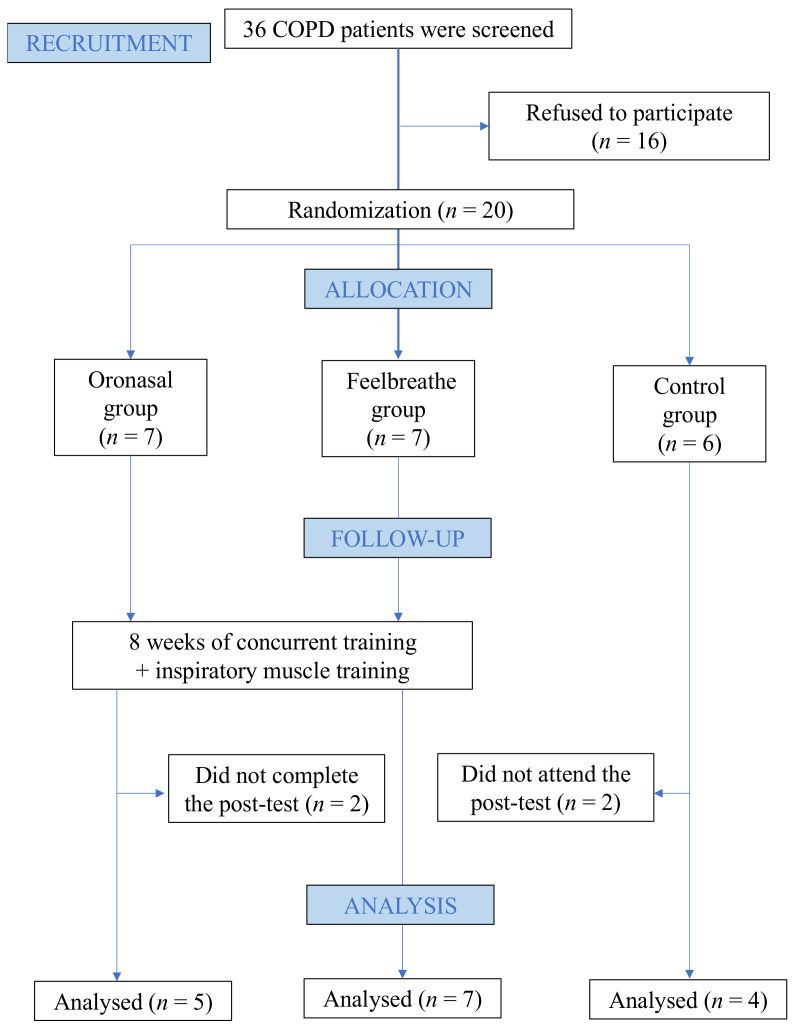
Flowchart of the study.

Thus, 36 patients were eligible for the study at the University Hospital Puerta del Mar (Cádiz, Spain), of whom 6 refused to participate and 10 did not meet one or more inclusion criteria. Two patients assigned to ONBG left the study during the final tests and two participants from CG were discarded for not attending the final tests and presenting exacerbated COPD. Finally, 16 individuals completed the study (Figure 1).

### 2.4. Training Programme

The participants of FBG and ONBG carried out a weekly 3-day training plan for 8 weeks following the exercise recommendations for COPD patients [20]. Each training session, with a total duration of 60 min, consisted in 15 min of general warm-up, followed by cardiorespiratory capacity improvement exercises, muscle-strengthening exercises and, lastly, respiratory exercises (see Supplementary Materials for a detailed description). Additionally, joint mobility and flexibility exercises were included.

The training session was divided into a warm-up phase, main phase and return to calmness, with the first two sessions being dedicated to the introduction to and familiarisation with the facilities, the trainer and the exercises. The participants were not asked to go for the next phase or exercise if they found it difficult to tolerate the previous phase or exercise. After each training session, the participants were subjected to a 1–10 perceived exertion scale, ensuring that scores of 8–9 were never reached.

CG did not carry out any programmed physical activity, i.e., the participants of the control group only performed the physical activity that is usually recommended by their pulmonologist.

The exercises for the improvement of the cardiorespiratory capacity were conducted at an intensity of 60–70% of the participant’s heart rate reserve, which was individually controlled using pulsometers (Polar Electro, Kempele, Finland) and measured through the perceived exertion scale [21]. Depending on the session, the exercise was performed on a running track, a treadmill or a stationary bicycle. The volume varied from 10 min in the first three sessions to 25 min in the last sessions.

For the strength exercises, we individually determined the intensity that allowed performing 3 sets of 8–12 repetitions in each of the exercises until reaching muscle fatigue. Once the participant was able to perform more than 12 repetitions for two consecutive training sessions, the intensity was increased by 2–5 kg, depending on the exercise, while maintaining the same volume. The exercises were performed with fitness machines for both upper and lower limbs.

The respiratory exercises, conducted at the end of each session, consisted in exercises with pursed lips, diaphragmatic, abdominal and thoracic breathing, and techniques of pulmonary expansion and rib cage mobilisation. The patients stretched the muscle groups that were worked out in each session.

**Figure 2:**
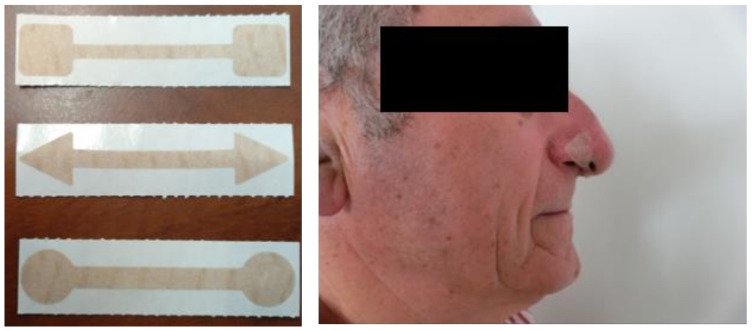
Types (**left**) and positioning (**right**) of the FeelBreathe device.

During the first training week, the FBG participants used the 4 mm FB device, which was replaced with a larger FB (5–6 mm) depending on the adaptation and improvement of the individual. The FB was placed under the nostrils using sterile gloves and ensuring that the participant did not have mucus or injuries (Figure 2). All participants, during the execution of the exercises, were asked to breathe correctly through nasal inspirations and mouth expirations.

All training sessions were directed by Graduates in Physical Activity and Sports Science and supervised by each participant’s specialist pulmonologist.

### 2.5. Cardiopulmonary Exercise Test

An incremental cardiopulmonary exercise test on a treadmill (Technogym Run Race 1400HC, Gambettola, Italy) was carried out by all participants before initiating the training programme and 2 days after its termination. The protocol for the realisation of the incremental test on a treadmill for COPD patients has been previously described [22,23]. The ventilatory variables and those of gas exchange were measured throughout the entire test using a gas analyser (Jaeger-CareFusion, MasterScreen CPX model, Hoechberg, Germany). The oxygen consumption (VO_2_), carbon dioxide production (VCO_2_), minute ventilation (VE), breathing frequency (BF), heart rate (HR), inspiratory tidal volume (VTin), expiratory tidal volume (VTex), inspiratory time (Tin), expiratory time (Tex), breathing cycle (TiTot), ventilatory equivalent of O_2_ (EqO_2_) and CO_2_ (EqCO_2_), end-tidal O_2_ and CO_2_ (PETO_2_ and PETCO_2_, respectively) and respiratory exchange rate (RER) were recorded respiration by respiration. Additionally, the total time of the test (T_t_) was recorded, and we calculated the lowest point in the curve that describes the relationship between VE and VCO_2_ (VE/VCO_2_ nadir) and the intercept and slope of the regression line that corresponded to the relationship between VE and VCO_2_ (VE/VCO_2_ intercept and VE/VCO_2_ slope, respectively). These variables represent the ventilatory efficiency of the patient during the cardiopulmonary exercise test and are related to the risk of mortality of the COPD patient [24,25,26].

VE/VCO_2_ intercept, intercept of the regression line VE/VCO_2_; VE/VCO_2_ nadir, lowest point in the curve that describes the relationship between VE/VCO_2_; VE/VCO_2_ slope, slope of the regression line VE/VCO_2_.

### 2.6. Statistical Analysis

**Figure 3:**
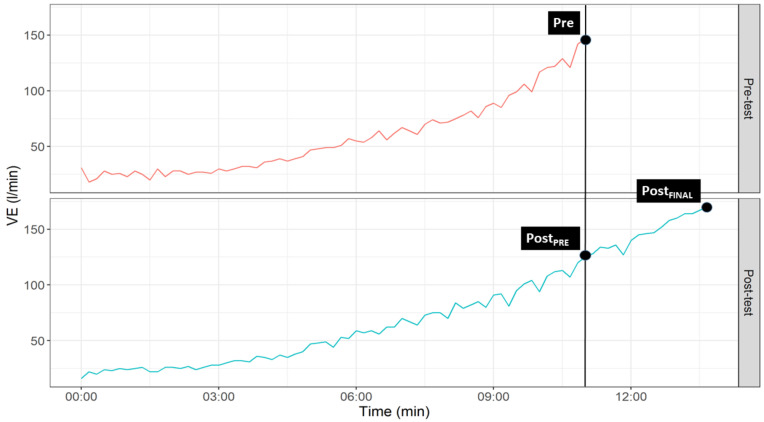
Graphical representation of temporal points selected for statistical analyses.

The results of this study are presented as mean ± standard deviation. For the analysis of the cardiorespiratory variables, we obtained the values of 3 different time points during the cardiopulmonary exercise test: the maximum value obtained during the pre-training test (Pre), the value obtained in the post-training test at the time when the maximum value in the pre-training test was obtained (Post_PRE_) and the maximum value recorded in the post-training test (Post_FINAL_) (Figure 3 shows an example with VE). This analysis was performed for each of training groups (i.e., FBG, ONBG and CG).

Intra- and inter-group differences were analysed using a Bayesian hierarchical regression model. For their estimation, all the hyper-parameters of the model had an a priori probability distribution with little information (i.e., a probability distribution that has enough information to restrict the range of values while leaving a wide range of uncovered values) [27]. The brms package for R programming language and data analysis was used to estimate the parameters of the model [28]. The Bayes factors were used to quantify the level of evidence of the results [29]. Additionally, the percentage of change between Pre and Post_PRE_ and between Pre and Post_FINAL_ was calculated using the formula: (Post value − Pre value)/Pre value * 100. Increments (Δ) from Pre to Post_PRE_ and Post_FINAL_ were calculated as mean and 95% highest density interval (HDI). HDI is a special case of credible interval which summarizes the uncertainty of the parameter estimated in such a way that any parameter value inside a 95%HDI are the 95% most credible values. All the results can be found in the supplementary materials (table format) while the significant results are displayed graphically in the results section.

## 3. Results

### 3.1. Descriptive Characteristics

The descriptive characteristics of the participants at the beginning of the study are shown in Table 1. ONBG obtained a lower FVC than CG (−665 mL (1317, 23), BF_10_ > 100).

### 3.2. Intra-Group Differences

**Figure 4:**
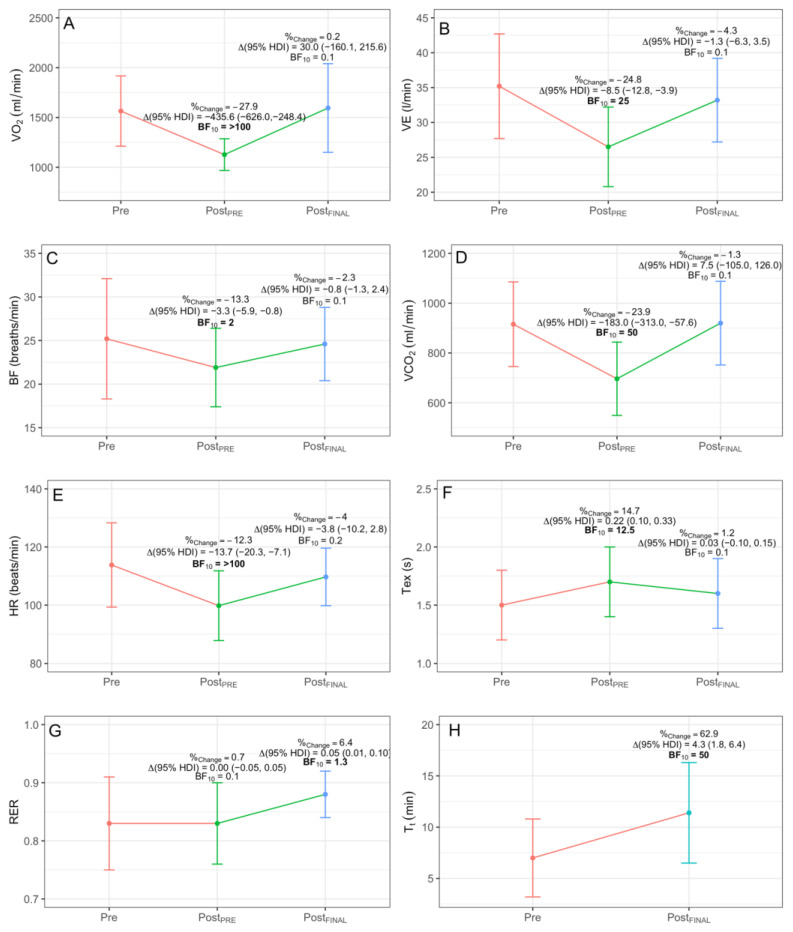
Significant intra-group differences for FBG in VO_2_ (**A**), VE (**B**), BF (**C**), VCO_2_ (**D**), HR (**E**), Tex (**F**), RER (**G**) and T_t_ (**H**). %_Change_ indicates percentage of change; Δ, increment; BF_10_, Bayes Factor; HDI, highest density interval. Significant differences are highlighted in **bold**.

**Figure 5:**
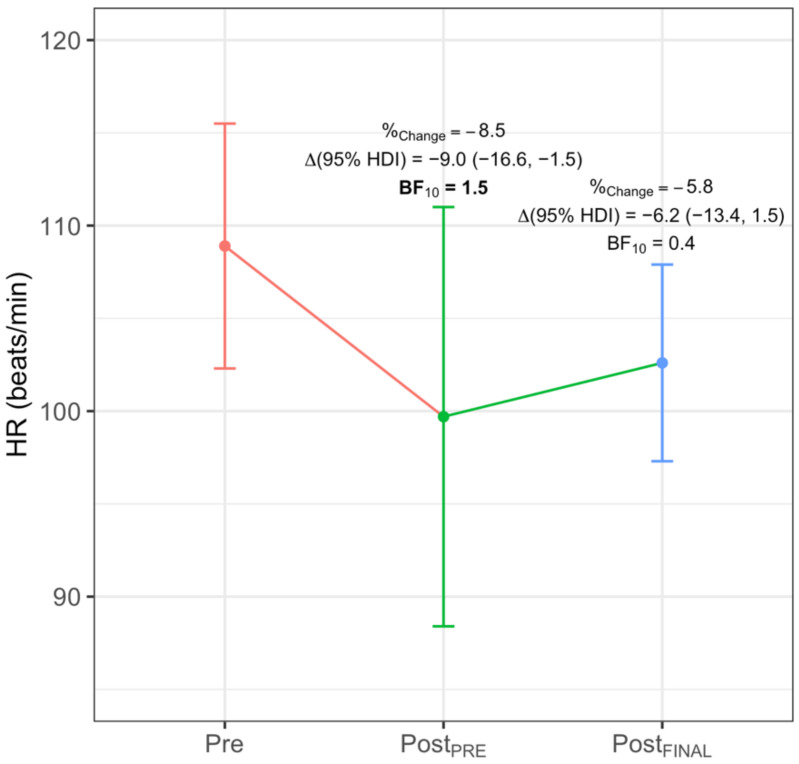
Significant intra-group difference for ONBG in HR. %_Change_ indicates percentage of change; Δ, increment; BF_10_, Bayes Factor; HDI, highest density interval. Significant differences are highlighted in **bold**.

**Figure 6:**
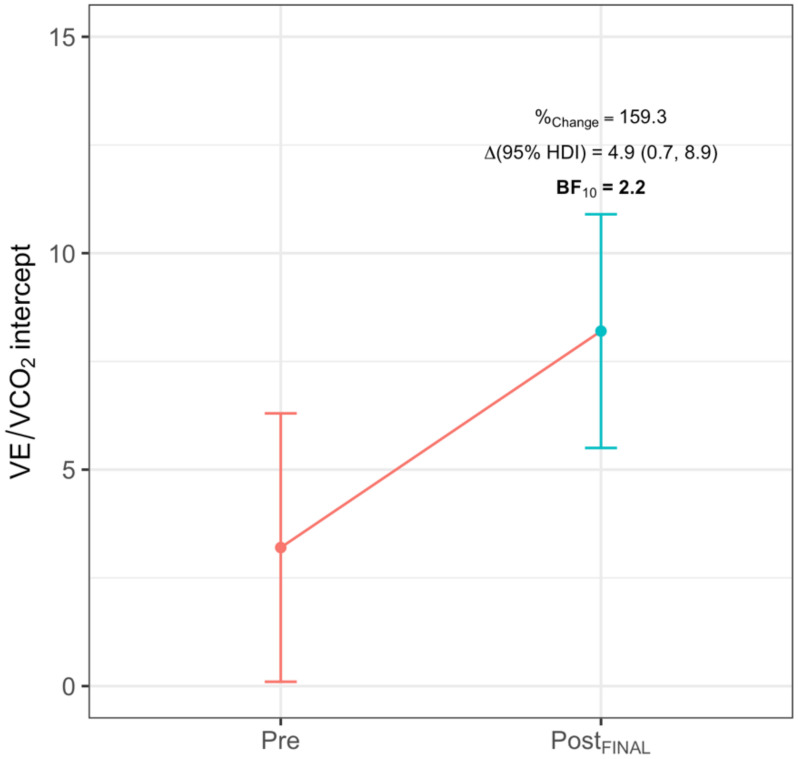
Significant intra-group difference for CG in VE/VCO_2_ intercept. %_Change_ indicates percentage of change; Δ, increment; BF_10_, Bayes Factor; HDI, highest density interval. Significant differences are highlighted in **bold**.

FBG (Figure 4) obtained lower Post_PRE_ values in VO_2_ (−435.6 mL/min (−626.0, −248.4), BF_10_ > 100), VE (−8.5 L/min (−12.8, −3.9), BF_10_ = 25), RR (−3.3 breaths/min (−5.9, −0.8), BF_10_ = 2), HR (−13.7 beats/min (−20.3, −7.1), BF_10_ > 100) and VCO_2_ (−183.0 L/min (−313.0, −57.6), BF_10_ = 50), and a greater value in Tex (0.22 s (0.10, 0.33), BF_10_ = 12.5). At Post_FINAL_, FBG obtained a greater value in T_t_ (4.3 min (1.8, 6.4), BF_10_ = 50) and RER (0.05 (0.01, 0.10), BF_10_ = 1.3). ONBG (Figure 5) showed a lower Post_PRE_ value in HR (−9.0 beats/min (−16.6, −1.5), BF_10_ = 1.5). Lastly, CG (Figure 6) obtained a greater value in the ordinate axis of VE/VCO_2_ (4.9 (0.7, 8.9), BF_10_ = 2.2).

### 3.3. Inter-Group Differences

**Figure 7:**
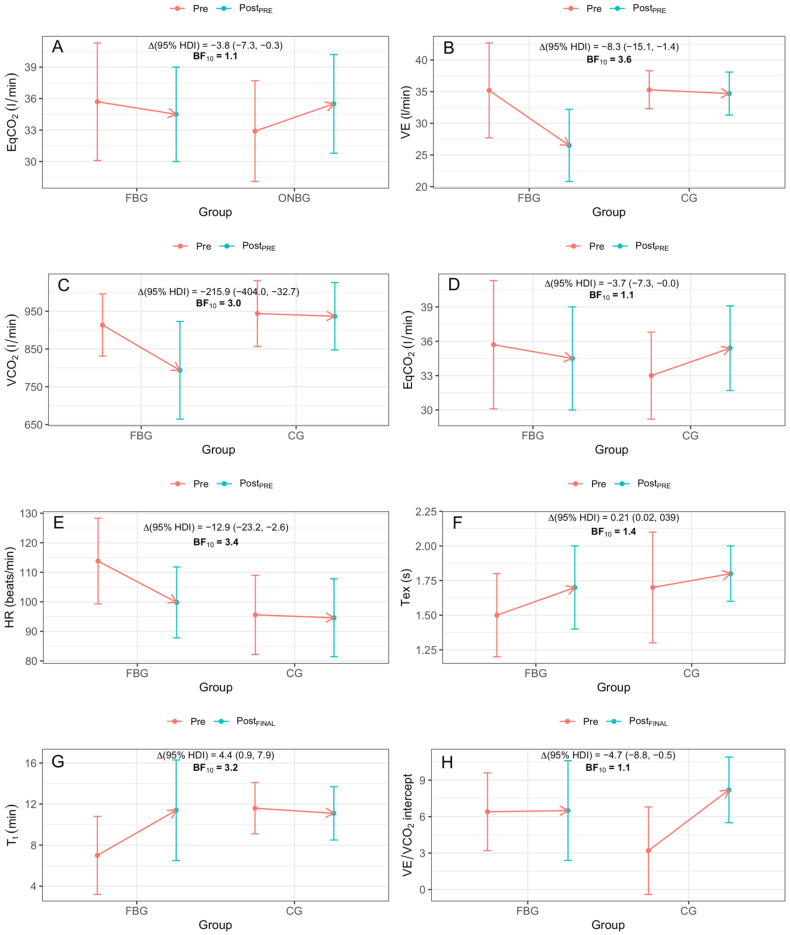
Significant inter-group differences between FBG and ONBG in EqCO_2_ at Post_PRE_ (**A**); between FBG and CG in VE (**B**), VCO_2_ (**C**), EqCO_2_ (**D**), HR (**E**) and Tex (**F**) at Post_PRE_; and between FBG and CG in T_t_ (**G**) and VE/VCO_2_ intercept (**H**). %_Change_ indicates percentage of change; Δ, increment; BF_10_, Bayes Factor; HDI, highest density interval. Significant differences are highlighted in **bold**.

The inter-group differences in the reached increments are shown in Figure 7. At Pre_POST_, FBG obtained a greater negative increment than ONBG in EqCO_2_ (−3.8 L/min (−7.3, −0.3), BF_10_ = 1.1) and compared to CG in VE (−8.3 L/min (−15.1, −1.4), BF_10_ = 3.6), VCO_2_ (−215.9 L/min (−404.0, −32.7), BF_10_ = 3.0), EqCO2 (−3.7 L/min (−7.3, −0.0), BF_10_ = 1.1) and HR (−12.9 beats/min (−23.2, −2.6), BF_10_ = 3.4). FBG also showed a greater Pre_POST_ positive increment in Tex (0.21 s (0.02, 0.39), BF_10_ = 1.4) with respect to CG.

At Post_FINAL_, FBG presented a greater positive increment compared to CG in T_t_ (4.4 min (0.9, 7.9), BF_10_ = 3.2) and negative in the VE/VCO_2_ intercept (−4.7 (−8.8, −0.5), BF_10_ = 1.1).

## 4. Discussion

To the best of the authors’ knowledge, this is the first study to consider IMT concurrently with exercise training in COPD patients. The main finding of this work is that, after 8 weeks of concurrent training of the cardiorespiratory capacity, muscle strength and IMT with FB, the COPD patients showed improvements in muscle dysfunction, exercise tolerance, physical performance and ventilatory efficiency. On the other hand, those participants who only performed the cardiorespiratory capacity and muscle strength training without IMT did not experience any relevant changes.

In general, the improvement in physical performance after the training in FBG is shown by the fact that they obtained a greater total time in the test (62.5% ↑), caused by an improvement in cardiopulmonary efficiency (i.e., lower VO_2_ for the same load). Lower VO_2_ (27.9% ↓) and HR (12.3% ↓) at Pre_POST_ could be due to a more efficient use of oxygen, after a decrease of the mitochondrial oxidative stress in the skeletal muscle [30,31]. Moreover, lower VE (24.8% ↓), RR (13.3% ↓) and VCO_2_ (23.9% ↓) and greater Tex (14.7% ↑) at Pre_POST_ indicate an improvement in ventilatory efficiency.

These changes were similar to, although slightly lower than, those reported by Casaburi et al. [32], who found a decrease in VO_2_ (6% ↓), HR (8% ↓), VE (15% ↓) and VCO_2_ (11% ↓) for the same exercise intensity after a 6-week pulmonary rehabilitation programme based on interval training with cycle ergometer. The difference in the magnitude of the changes can be due to the fact that the participants only trained the cardiorespiratory capacity (although at high intensity), they did not perform concurrent IMT training and the duration of the mentioned study was shorter.

In this study there was an improvement in many of the analyzed parameters in the FBG compare with the ONBG at Post_FINAL_ but they did not reach statistical significance, probably due to sample size, however, the FBG reached 2.1 min more that the ONBG at the end of the cardiopulmonary exercise test what is more than 100 s that is considered as the minimal clinical important difference [33]. VO_2_, VE and EqCO_2_ were reduced in the Post_PRE_, being the EqCO_2_ statistically significant. These findings indicate that patients in the FBG only showed one statically significant difference in the respiratory response compared to the ONBG. Therefore, more studies are necessary to reach a definitive conclusion about including FB in a pulmonary rehabilitation programme.

Recent studies have not found an additive effect of IMT in a pulmonary rehabilitation programme on the capacity to perform physical exercise [7,8,9]. Wang et al. [9] reported that there were no statistically significant differences between those participants who carried out 8-week aerobic training on a cycle ergometer added to an IMT and those who only conducted the aerobic training, although both groups obtained a significant improvement in the capacity to perform exercise. Similarly, Beaumont et al. [7] did not find differences at the end of a 4-week training between groups in the 6 min walk test (6MWT). Lastly, Schultz et al. [8], despite analysing a large sample of COPD patients (*n* = 561), only conducted the experiment for 3 weeks, concluding that IMT did not provide an additional gain in the physical capacity measured through the 6MWT. These results could be partly because the IMT was carried out in addition to the training sessions, using a threshold device, which cannot be used concurrently with exercise. Therefore, the use of devices such as FB for IMT during physical exercise may be a burden that could adequately induce respiratory muscle fatigue and cause adaptations for the improvement of physical performance [34,35,36].

Regarding the execution of strength exercises, it was observed that both FBG and ONBG ostensibly improved their capacity to mobilise loads, although the real improvement of this capacity is unknown, since initial and final tests about the maximum mobilised load were not conducted. Similar programmes with strength exercises have been applied in COPD patients, thus it is recommended to include it in respiratory rehabilitation programmes. The effect of FB during the execution of these exercises, which involve maximum inspirations and expirations, could have influenced the fact that FBG obtained better results than ONBG.

This study has several limitations, with the main one being the small sample size and the final sample imbalance of the groups. To solve this problem, we applied a Bayesian inference process (i.e., quantifying the uncertainty about a given amount based on the available data and previous information), incorporating information about the parameters of interest to the a priori distributions to obtain a reliable estimation of such values. Another limitation was that, due to the characteristics of the FB device, it was not possible to perform a double-blind design for the intervention. Lastly, the sample was only constituted by men, thus the results obtained cannot be extrapolated to the women.

## 5. Conclusions

The use of the FB device during a pulmonary rehabilitation programme improves exercise tolerance and ventilatory efficiency and induces changes in the ventilatory pattern that could lead to a reduction of the dynamic hyperinflation. This simple device, known as FB, could be a useful tool for the IMT of COPD patients outside of the hospital or health centre, since it can be worn while performing activities of daily living.

## Figures and Tables

**Table 1:** Descriptive characteristics of the sample.

Variables	FBG (*n* = 7)	ONBG (*n* = 5)	CG (*n* = 4)	FBG vs. ONBG	BF_10_	FBG vs. CG	BF_10_	ONBG vs. CG	BF_10_
Age (years)	65.0 ± 8.0	72.0 ± 7.4	70.2 ± 5.9	−4.8 (−14.8, 6.2)	0.1	−6.6 (−15.5, 3.6)	0.3	1.7 (−10.0, 12.9)	0.1
BMI (m/kg^2^)	28.4 ± 4.2	26.8 ± 2.5	25.9 ± 2.1	1.5 (−2.4, 5.4)	0.3	2.3 (−2.0, 6.3)	0.2	0.8 (−3.6, 5.5)	0.2
FEV_1_ (mL)	1571 ± 334	1608 ± 344	1812 ± 706	−23.5 (−557, 469)	0.1	−218 (−760, 349)	0.1	194 (−441, 773)	0.1
FEV_1_ (% predicted)	46.9 ± 10.6	51.2 ± 9.8	52.6 ± 19.9	−3.7 (−18.4, 11.1)	0.1	−5.4 (−21.6, 10.8)	0.1	−1.8 (−19.8, 16.1)	0.1
FVC (mL)	2869 ± 298	2580 ± 577	3270 ± 474	283 (−260, 801)	0.3	−382 (−929, 207)	0.1	**−665 (−1317, −22.9)**	**>100**
FVC (% predicted)	63.9 ± 8.3	59.2 ± 10.0	67.1 ± 13.8	4.5 (−7.4, 17.1)	0.3	−3.1 (−15.9, 10.1)	0.1	−7.6 (−21.5, 7.7)	0.3
FEV/FVC (%)	54.1 ± 6.9	62.6 ± 5.6	54.2 ± 14.7	−8.1 (−18.7, 2.8)	0.2	0.0 (−11.4, 11.4)	0.1	8.1 (−4.2, 20.8)	0.3
P_Imax_ (mmHg)	93.3 ± 19.1	85.6 ± 23.9	102.0 ± 14.9	7.9 (−15.6, 31.1)	0.1	−7.6 (−34.3, 17.3)	0.2	−15.5 (−42.8, 12.5)	0.2
CAT (score)	9.7 ± 6.5	10.0 ± 4.5	6.8 ± 4.4	−0.3 (−6.8, 6.4)	0.1	2.7 (−3.8, 9.7)	0.2	3.0 (−4.5, 10)	0.1
mMRC (score|%)				P(Y|FB)		P(Y|ONB)		P(Y|CG)	
0	0 (0%)	0 (0%)	0 (0%)	0.0 (0.0, 0.0)		0.0 (0.0, 0.0)		0.0 (0.0, 0.0)	
1	0 (0%)	0 (0%)	0 (0%)	0.0 (0.0, 0.1)		0.0 (0.0, 0.2)		0.0 (0.0, 0.2)	
2	6 (85%)	5 (100%)	4 (100%)	0.9 (0.6, 0.1)		0.9 (0.7, 1.0)		0.9 (0.7, 1.0)	
3	1 (15%)	0 (0%)	0 (0%)	0.1 (0.0, 0.4)		0.0 (0.0, 0.2)		0.0 (0.0, 0.3)	

FBG, training + breathing with FeelBreathe group; ONBG, training + breathing without FeelBreathe group; CG, control group; BMI, body mass index; FEV_1_, forced expiratory volume in 1 s; FVC, forced vital capacity; HDI, highest density interval; P_Imax_, maximal inspiratory pressure; CAT, COPD Assessment Test; mMRC, modified Medical Research Council dyspnea scale; P(Y|FB), P(Y|ONB) and P(Y|GC), probability of answer the score Y (i.e., 0, 1, 2, 3), given the participant was assigned to a group at baseline. 95% highest density intervals that does not include 0 for continuous variables or 0.5 for categorical variables are highlighted in **bold**.

## Data Availability

The dataset analyzed in this study can be found in https://github.com/JorgeDelro/COPD (accessed on 7 January 2021).

## Supplementary Materials

The following are available online at https://www.mdpi.com/article/10.3390/ijerph18084207/s1, Table S1. Mean maximum value obtained by the Feelbreathe group (FBG) in each variable in the pre-training test (Pre-Value), value obtained in the post-training test at the time when the maximum value in the pre-training test was obtained (Post_PRE_ – Value) and maximum value obtained in the post-training test (Post_FINAL_ – Value). Table S2: Mean maximum value obtained by the oronasal breathing group (ONBG) in each variable in the pre-training test (Pre-Value), value obtained in the post-training test at the time when the maximum value in the pre-training test was obtained (Post_PRE_ – Value) and maximum value obtained in the post-training test (Post_FINAL_ – Value). Table S3: Mean of the maximum value obtained by the control group (CG) in each variable in the pre-training test (Pre-Value) and maximum value obtained in the post-training test (Post_FINAL_ – Value).

## Abbreviations

BF_10_:Bayes factor; BF, breathing frequency; CAT, COPD Assessment Test; EqO2, ventilatory equivalent of O_2_; EqCO_2_, ventilatory equivalent of CO_2_; FEV1, forced expiratory volume in 1 s; FVC, forced vital capacity; HDI, highest density interval; mMRC, modified Medical Research Council dyspnea scale; PETCO_2_, end-tidal CO_2_; PETO_2_, end-tidal O_2_; P_Imax_, maximal inspiratory pressure; Pre, pre-training test; Post_PRE_, value obtained in the post-training test at the time when the maximum value in the pre-training test was obtained; Post_FINAL_, maximum value obtained in the post-training test; RER, respiratory exchange rate; Tex, expiratory time; Tin, inspiratory time; TiTot, breathing cycle; T_t_, total test time; VCO_2_, carbon dioxide production; VE, minute ventilation; VE/VCO_2_ intercept, intercept of the regression line VE/VCO_2_; VE/VCO_2_ nadir, lowest point in the curve that describes the relationship VE/VCO_2_; VE/VCO_2_ slope, slope of the regression line VE/VCO_2_; Vtex, expiratory tidal volume; VO_2_, oxygen consumption; Vtin, inspiratory tidal volume.

## References

[1] Rabe K.F., Watz H. (2017). Chronic Obstructive Pulmonary Disease. Lancet.

[2] Vogelmeier C.F., Criner G.J., Martinez F.J., Anzueto A., Barnes P.J., Bourbeau J., Celli B.R., Chen R., Decramer M., Fabbri L.M. (2017). Global Strategy for the Diagnosis, Management, and Prevention of Chronic Obstructive Lung Disease 2017 Report. GOLD Executive Summary. Am. J. Respir. Crit. Care Med..

[3] McCarthy B., Casey D., Devane D., Murphy K., Murphy E., Lacasse Y. (2015). Pulmonary Rehabilitation for Chronic Obstructive Pulmonary Disease. Cochrane Database Syst. Rev..

[4] Garvey C., Bayles M.P., Hamm L.F., Hill K., Holland A., Limberg T.M., Spruit M.A. (2016). Pulmonary Rehabilitation Exercise Prescription in Chronic Obstructive Pulmonary Disease: Review of Selected Guidelines: An Official Statement from the American Association of Cardiovascular and Pulmonary Rehabilitation. J. Cardiopulm. Rehabil. Prev..

[5] Gosselink R., De Vos J., van den Heuvel S.P., Segers J., Decramer M., Kwakkel G. (2011). Impact of Inspiratory Muscle Training in Patients with COPD: What Is the Evidence?. Eur. Respir. J..

[6] Beaumont M., Forget P., Couturaud F., Reychler G. (2018). Effects of Inspiratory Muscle Training in COPD Patients: A Systematic Review and Meta-Analysis. Clin. Respir. J..

[7] Beaumont M., Mialon P., Le Ber C., Le Mevel P., Péran L., Meurisse O., Morelot-Panzini C., Dion A., Couturaud F. (2018). Effects of Inspiratory Muscle Training on Dyspnoea in Severe COPD Patients during Pulmonary Rehabilitation: Controlled Randomised Trial. Eur. Respir. J..

[8] Schultz K., Jelusic D., Wittmann M., Krämer B., Huber V., Fuchs S., Lehbert N., Wingart S., Stojanovic D., Göhl O. (2018). Inspiratory Muscle Training Does Not Improve Clinical Outcomes in 3-Week COPD Rehabilitation: Results from a Randomised Controlled Trial. Eur. Respir. J..

[9] Wang K., Zeng G.-Q., Li R., Luo Y.-W., Wang M., Hu Y.-H., Xu W.-H., Zhou L.-Q., Chen R.-C., Chen X. (2017). Cycle Ergometer and Inspiratory Muscle Training Offer Modest Benefit Compared with Cycle Ergometer Alone: A Comprehensive Assessment in Stable COPD Patients. Int. J. Chron. Obstruct. Pulmon. Dis..

[10] Shei R.-J. (2018). Recent Advancements in Our Understanding of the Ergogenic Effect of Respiratory Muscle Training in Healthy Humans: A Systematic Review. J. Strength Cond. Res..

[11] Gonzalez-Montesinos J.L., Arnedillo A., Vaz-Pardal C., Fernandez-Santos J.R. Dispositivo Para El Entrenamiento de La Musculatura Nasal. Utility model U201930922, 6 August 2019. https://consultas2.oepm.es/InvenesWeb/detalle?referencia=PCT/ES2020/070364.

[12] Gonzalez-Montesinos J.L., Arnedillo A., Fernandez-Santos J.R., Vaz-Pardal C., García P.A., Castro-Piñero J., Ponce-González J.G. (2020). A New Nasal Restriction Device Called FeelBreathe(^®^) Improves Breathing Patterns in Chronic Obstructive Pulmonary Disease Patients during Exercise. Int. J. Environ. Res. Public Health.

[13] Arnedillo A., Gonzalez-Montesinos J.L., Fernandez-Santos J.R., Vaz-Pardal C., España-Domínguez C., Ponce-González J.G., Cuenca-García M. (2020). Effects of a Rehabilitation Programme with a Nasal Inspiratory Restriction Device on Exercise Capacity and Quality of Life in COPD. Int. J. Environ. Res. Public Health.

[14] Boutou A.K., Zafeiridis A., Pitsiou G., Dipla K., Kioumis I., Stanopoulos I. (2020). Cardiopulmonary Exercise Testing in Chronic Obstructive Pulmonary Disease: An Update on Its Clinical Value and Applications. Clin. Physiol. Funct. Imaging.

[15] Stringer W., Marciniuk D. (2018). The Role of Cardiopulmonary Exercise Testing (CPET) in Pulmonary Rehabilitation (PR) of Chronic Obstructive Pulmonary Disease (COPD) Patients. COPD.

[16] Palange P., Ward S.A., Carlsen K.-H., Casaburi R., Gallagher C.G., Gosselink R., O’Donnell D.E., Puente-Maestu L., Schols A.M., Singh S. (2007). Recommendations on the Use of Exercise Testing in Clinical Practice. Eur. Respir. J..

[17] Radtke T., Crook S., Kaltsakas G., Louvaris Z., Berton D., Urquhart D.S., Kampouras A., Rabinovich R.A., Verges S., Kontopidis D. (2019). ERS Statement on Standardisation of Cardiopulmonary Exercise Testing in Chronic Lung Diseases. Eur. Respir. Rev..

[18] Uschner D., Schindler D., Hilgers R.-D., Heussen N. (2018). RandomizeR: An R Package for the Assessment and Implementation of Randomization in Clinical Trials. J. Stat. Softw..

[19] Bestall J.C., Paul E.A., Garrod R., Garnham R., Jones P.W., Wedzicha J.A. (1999). Usefulness of the Medical Research Council (MRC) Dyspnoea Scale as a Measure of Disability in Patients with Chronic Obstructive Pulmonary Disease. Thorax.

[20] Gloeckl R., Marinov B., Pitta F. (2013). Practical Recommendations for Exercise Training in Patients with COPD. Eur. Respir. Rev..

[21] Borg G.A. (1982). Psychophysical Bases of Perceived Exertion. Med. Sci. Sports Exerc..

[22] Hsia D., Casaburi R., Pradhan A., Torres E., Porszasz J. (2009). Physiological Responses to Linear Treadmill and Cycle Ergometer Exercise in COPD. Eur. Respir. J..

[23] Holm S.M., Rodgers W., Haennel R.G., MacDonald G.F., Bryan T.L., Bhutani M., Wong E., Stickland M.K. (2014). Effect of Modality on Cardiopulmonary Exercise Testing in Male and Female COPD Patients. Respir. Physiol. Neurobiol..

[24] Neder J.A., Berton D.C., Arbex F.F., Alencar M.C., Rocha A., Sperandio P.A., Palange P., O’Donnell D.E. (2017). Physiological and Clinical Relevance of Exercise Ventilatory Efficiency in COPD. Eur. Respir. J..

[25] Neder J.A., Alharbi A., Berton D.C., Alencar M.C.N., Arbex F.F., Hirai D.M., Webb K.A., O’Donnell D.E. (2016). Exercise Ventilatory Inefficiency Adds to Lung Function in Predicting Mortality in COPD. COPD.

[26] Phillips D.B., Collins S.É., Stickland M.K. (2020). Measurement and Interpretation of Exercise Ventilatory Efficiency. Front. Physiol..

[27] Gelman A., Simpson D., Betancourt M. (2017). The Prior Can Generally Only Be Understood in the Context of the Likelihood. Entropy.

[28] Bürkner P.-C. (2017). Brms: An R Package for Bayesian Multilevel Models Using Stan. J. Stat. Softw..

[29] Wagenmakers E.-J., Lodewyckx T., Kuriyal H., Grasman R. (2010). Bayesian Hypothesis Testing for Psychologists: A Tutorial on the Savage-Dickey Method. Cogn. Psychol..

[30] Calvert L.D., Singh S.J., Morgan M.D., Steiner M.C. (2011). Exercise Induced Skeletal Muscle Metabolic Stress Is Reduced after Pulmonary Rehabilitation in COPD. Respir. Med..

[31] Maltais F., LeBlanc P., Simard C., Jobin J., Bérubé C., Bruneau J., Carrier L., Belleau R. (1996). Skeletal Muscle Adaptation to Endurance Training in Patients with Chronic Obstructive Pulmonary Disease. Am. J. Respir. Crit. Care Med..

[32] Casaburi R., Patessio A., Ioli F., Zanaboni S., Donner C.F., Wasserman K. (1991). Reductions in Exercise Lactic Acidosis and Ventilation as a Result of Exercise Training in Patients with Obstructive Lung Disease. Am. Rev. Respir. Dis..

[33] Güell Rous M.R., Díaz Lobato S., Rodríguez Trigo G., Morante Vélez F., San Miguel M., Cejudo P., Ortega Ruiz F., Muñoz A., Galdiz Iturri J.B., García A. (2014). Pulmonary rehabilitation. Sociedad Española de Neumología y Cirugía Torácica (SEPAR). Arch. Bronconeumol..

[34] Shei R.-J., Mickleborough T.D. (2019). Unresolved Questions That Need to Be Addressed in Order to Maximize the Efficacy of Inspiratory Muscle Training. Phys. Ther. Sport.

[35] Shei R.-J. (2020). Training Load Influences the Response to Inspiratory Muscle Training. J. Sports Sci. Med..

[36] Karsten M., Ribeiro G.S., Esquivel M.S., Matte D.L. (2019). Maximizing the Effectiveness of Inspiratory Muscle Training in Sports Performance: A Current Challenge. Phys. Ther. Sport.

