# Supplementary material for: Effects of a Rehabilitation Programme Using a Nasal Inspiratory Restriction Device in COPD
Source: Int J Environ Res Public Health. 2021 Apr 15;18(8):4207. doi: 10.3390/ijerph18084207 (PMC8071399; doi:10.3390/ijerph18084207)
Supplement: Supplementary file 1 [file ijerph-18-04207-s001.zip › ijerph-1130029-supplementary.pdf]

International Journal of
Environmental Research and Public Health

MDPI

Supplementary Material

Table S1. Mean maximum value obtained by the Feelbreathe group (FBG) in each variable in the pre-training test (Pre-Value), value obtained in the post-training test at the time when the maximum value in the pre-training test was obtained ($Post_{PRE}$ – Value) and maximum value obtained in the post-training test ($Post_{FINAL}$ – Value). The change percentage ($\%_{Change}$), increment (Δ) and 95% HDI are given for the changes from Pre to $Post_{PRE}$ and from Pre to $Post_{FINAL}$.

	FBG (*n* = 7)								
	Pre	$Post_{PRE}$				$Post_{FINAL}$			
Variable	Mean ± sd	Mean ± sd	$\%_{Change}$	Δ Mean (95% HDI)	BF_{10}	Mean ± sd	$\%_{Change}$	Δ Mean (95% HDI)	BF_{10}
T_t (min)	7.0 ± 3.8					**11.4 ± 4.9**	**62.9**	**4.3 (1.8, 6.4)**	**50**
VE/VCO_2 nadir	32.2 ± 4.8					29.4 ± 2.8	−8.7	−2.7 (−5.6, 0.3)	0.5
VE/VCO_2 slope	31.0 ± 5.1					28.6 ± 4.6	−7.7	−2.4 (−6.5, 1.7)	0.3
VE/VCO_2 intercept	6.4 ± 3.2					6.5 ± 4.1	2.0	0.2 (−3.2, 3.6)	0.1
VO_2 (ml/min)	1564.7±352.8	**1127.2 ± 159.3**	**−27.9**	**−435.6 (−626.0, −248.4)**	**>100**	1594.9 ± 445.1	0.2	30.0 (−160.1, 215.6)	0.1
VE (l/min)	35.2 ± 7.5	**26.5 ± 5.7**	**−24.8**	**−8.5 (−12.8, −3.9)**	**25**	33.2 ± 6.0	−4.3	−1.3 (−6.3, 3.5)	0.1
BF (breaths/min)	25.2 ± 6.9	**21.9 ± 4.5**	**−13.3**	**−3.3 (−5.9, −0.8)**	**2**	24.6 ± 4.2	−2.3	−0.8 (−1.3, 2.4)	0.1
VCO_2 (ml/min)	915.5 ± 169.8	**696.5 ± 147.3**	**−23.9**	**−183.0 (−313.0, −57.6)**	**50**	919.8 ± 168.1	−1.3	7.5 (−105.0, 126.0)	0.1
EqO_2 (l/min)	28.5 ± 3.0	29.2 ± 5.0	2.2	0.9 (−1.5, 3.0)	0.2	29.7 ± 4.0	3.5	1.3 (−1.1, 3.7)	0.1
$EqCO_2$ (l/min)	35.7 ± 5.6	34.5 ± 4.5	−3.4	−1.3 (−3.9, 1.4)	0.2	33.7 ± 4.0	−0.6	−2.0 (−4.8, 1.0)	0.2

HR (beats/min)	113.8 ± 14.5	**99.8 ± 12.0**	**−12.3**	**−13.7 (−20.3, −7.1)**	**>100**	109.7 ± 9.9	−4.0	−3.8 (−10.2, 2.8)	0.2
VTin (ml)	1.4 ± 0.2	1.3 ± 0.2	−6.1	−0.08 (−0.21, 0.04)	0.2	1.4 ± 0.2	−1.2	0.03 (−0.08, 0.14)	0.1
VTex (ml)	1.4 ± 0.2	1.2 ± 0.3	−9.4	−0.12 (−0.24, 0.02)	0.2	1.4 ± 0.2	−1.7	−0.02 (−0.12, 0.08)	0.1
Tin (s)	1.1 ± 0.4	1.1 ± 0.2	3.4	0.06 (−0.13, 0.27)	0.1	1.0 ± 0.2	0.0	−0.03 (−0.24, 0.17)	0.1
Tex (s)	1.5 ± 0.3	**1.7 ± 0.3**	**14.7**	**0.22 (0.10, 0.33)**	**12.5**	1.6 ± 0.3	1.2	0.03 (−0.10, 0.15)	0.1
TiTot (%)	40.0 ± 5.0	38.7 ± 4.8	−3.6	−1.32 (−4.13, 1.47)	0.1	39.2 ± 4.3	−0.5	−0.65 (−3.16, 1.72)	0.1
$PETO_2$ (mmHg)	14.8 ± 0.3	14.7 ± 0.6	−0.2	−0.03 (−0.23, 0.23)	0.1	14.8 ± 0.5	−0.8	−0.00 (−0.29, 0.27)	0.1
$PETCO_2$ (mmHg)	4.3 ± 0.4	4.3 ± 0.3	0.3	0.00 (−0.20, 0.20)	0.1	4.5 ± 0.3	3.5	0.16 (−0.13, 0.45)	0.2
RER	0.83 ± 0.08	0.83 ± 0.07	0.7	0.00 (−0.05, 0.05)	0.1	**0.88 ± 0.04**	**6.4**	**0.05 (0.01, 0.10)**	**1.3**

BF_{10}, Bayes factor; BF, breathing frequency; EqO2, ventilatory equivalent of O_2; $EqCO_2$, ventilatory equivalent of CO_2; HDI, highest density interval; $PETCO_2$, end-tidal CO_2; $PETO_2$, end-tidal O_2; Pre, pre-training test; $Post_{PRE}$, value obtained in the post-training test at the time when the maximum value in the pre-training test was obtained; $Post_{FINAL}$, maximum value obtained in the post-training test; RER, respiratory exchange rate; Tex, expiratory time; Tin, inspiratory time; TiTot, breathing cycle; T_t, total test time; VCO_2, carbon dioxide production; VE, minute ventilation; VE/VCO_2 intercept, intercept of the regression line VE/VCO_2; VE/VCO_2 nadir, lowest point in the curve that describes the relationship VE/VCO_2; VE/VCO_2 slope, slope of the regression line VE/VCO_2; Vtex, expiratory tidal volume; VO_2, oxygen consumption; Vtin, inspiratory tidal volume. 95% highest density intervals that does not include 0 are highlighted in **bold.**

Table S2. Mean maximum value obtained by the oronasal breathing group (ONBG) in each variable in the pre-training test (Pre-Value), value obtained in the post-training test at the time when the maximum value in the pre-training test was obtained ($Post_{PRE}$ – Value) and maximum value obtained in the post-training test ($Post_{FINAL}$ – Value). The change percentage ($\%_{Change}$), increment (Δ) and 95% HDI are given for the changes from Pre to $Post_{PRE}$ and from Pre to $Post_{FINAL}$.

	ONBG (n = 5)								
	Pre	$Post_{PRE}$				**$Post_{FINAL}$**			
Variable	Mean ± sd	Mean ± sd	$\%_{Change}$	Δ Mean (95% HDI)	BF_{10}	Mean ± sd	$\%_{Change}$	Δ Mean (95% HDI)	BF_{10}
T_t (min)	7.5 ± 3.8					9.9 ± 1.8	31.7	2.4 (−0.0, 5.1)	0.8
VE/VCO_2 nadir	31.2 ± 2.8					30.7 ± 1.8	−1.6	−0.2 (−3.9, 3.6)	0.1
VE/VCO_2 slope	32.4 ± 2.4					30.4 ± 4.0	−6.2	−1.2 (−6.0, 4.1)	0.3
VE/VCO_2 inter-cept	4.1 ± 2.8					5.8 ± 5.6	41.7	1.8 (−2.3, 6.0)	0.3
VO_2 (ml/min)	1485.0 ± 379.3	1262.3 ± 190.4	−15.0	−223.6 (−444.0, 0.1)	0.7	1583.8 ± 378.1	6.7	94.2 (−14.1 to 209.0)	0.1
VE (l/min)	32.5 ± 2.9	30.3 ± 3.2	−6.7	−2.2 (−7.5, 3.0)	0.2	32.6 ± 2.6	0.3	0.1 (−5.1, 5.7)	0.1
BF (breaths/min)	24.4 ± 3.5	24.0 ± 2.4	−1.8	−0.4 (−3.3, 2.7)	0.1	24.3 ± 3.5	−0.1	0.0 (−2.9, 2.8)	0.1
VCO_2 (ml/min)	913.5 ± 82.2	793.6 ± 129.3	−13.1	−115.0 (−251.9, 19.0)	0.5	897.6 ± 108.1	−1.7	−13.6 (−149.2, 122.5)	0.1
EqO_2 (l/min)	28.1 ± 5.5	33.9 ± 4.7	5.1	1.5 (−0.8, 3.7)	0.2	28.8 ± 5.6	2.2	0.6 (−1.6, 2.9)	0.1
$EqCO_2$ (l/min)	32.9 ± 4.8	35.5 ± 4.7	8.0	2.6 (−0.5, 5.3)	0.7	34.1 ± 4.9	3.8	1.3 (−1.5, 4.0)	0.2
HR (beats/min)	108.9 ± 6.6	**99.7 ± 11.3**	**−8.5**	**−9.0 (−16.6, −1.5)**	**1.5**	102.6 ± 5.3	−5.8	−6.2 (−13.4, 1.5)	0.4
VTin (ml)	1.4 ± 0.3	1.3 ± 0.2	−8.4	−0.12 (−0.24, 0.04)	0.3	1.3 ± 0.2	−4.1	−0.06 (−0.18, 0.06)	0.1

VTex (ml)	1.4 ± 0.2	1.3 ± 0.2	−5.6	−0.08 (−0.19, 0.04)	0.1	1.4 ± 0.2	−0.7	−0.01 (−0.13, 0.11)	0.1
Tin (s)	1.1 ± 0.2	1.0 ± 0.1	−3.0	−0.03 (−0.23, 0.16)	0.1	1.0 ± 0.2	0.0	−0.01 (−0.23, 0.22)	0.1
Tex (s)	1.5 ± 0.2	1.6 ± 0.2	2.0	0.03 (−0.10, 0.17)	0.1	1.5 ± 0.2	−2.0	−0.03 (−0.16, 0.11)	0.1
TiTot (%)	40.4 ± 2.9	38.5 ± 3.71	−4.7	−1.95 (−4.77, 0.86)	0.2	40.7 ± 2.5	0.8	0.26 (−2.56, 3.06)	0.1
$PETO_2$ (mmHg)	14.8 ± 0.8	14.8 ± 0.4	−0.4	−0.06 (−0.35, 0.22)	0.1	14.6 ± 0.8	−1.4	−0.21 (−0.49, 0.08)	0.2
$PETCO_2$ (mmHg)	4.5 ± 0.4	4.2 ± 0.4	−2.7	−0.12 (−0.35, 0.13)	0.1	4.5 ± 0.5	−0.2	−0.00 (−0.25, 0.23)	0.1
RER	0.85 ± 0.06	0.83 ± 0.05	−3.1	−0.02 (−0.08, 0.03)	0.2	0.84 ± 0.05	−1.4	−0.01 (−0.06, 0.05)	0.1

BF_{10}, Bayes factor; BF, breathing frequency; EqO2, ventilatory equivalent of O_2; $EqCO_2$, ventilatory equivalent of CO_2; HDI, highest density interval; $PETCO_2$, end-tidal CO_2; $PETO_2$, end-tidal O_2; Pre, pre-training test; $Post_{PRE}$, value obtained in the post-training test at the time when the maximum value in the pre-training test was obtained; $Post_{FINAL}$, maximum value obtained in the post-training test; RER, respiratory exchange rate; Tex, expiratory time; Tin, inspiratory time; TiTot, breathing cycle; T_t, total test time; VCO_2, carbon dioxide production; VE, minute ventilation; VE/VCO_2 intercept, intercept of the regression line VE/VCO_2; VE/VCO_2 nadir, lowest point in the curve that describes the relationship VE/VCO_2; VE/VCO_2 slope, slope of the regression line VE/VCO_2; Vtex, expiratory tidal volume; VO_2, oxygen consumption; Vtin, inspiratory tidal volume. 95% highest density intervals that does not include 0 are highlighted in **bold.**

Table S3. Mean of the maximum value obtained by the control group (CG) in each variable in the pre-training test (Pre-Value) and maximum value obtained in the post-training test ($Post_{FINAL}$ – Value). The percentage of change ($\%_{Change}$), increment (Δ) and 95% HDI are given for the changes from Pre to $Post_{FINAL}$.

	CG (n=4)				
Variable	Pre	$Post_{Final}$			
	Mean ± sd	Mean ± sd	$\%_{Change}$	Δ Mean (95% HDI)	BF_{10}
T_t (min)	11.6 ± 2.5	11.1 ± 2.6	-4.2	-0.5 (-3.4, 2.3)	0.1
VE/VCO_2 nadir	29.8 ± 4.3	30.7 ± 5.6	3.0	0.9 (-4.1, 2.6)	0.1
VE/VCO_2 slope	32.5 ± 8.0	27.5 ± 3.4	-15.4	-4.7 (-9.3, 0.3)	1.1
VE/VCO_2 intercept	3.2 ± 3.6	8.2 ± 2.7	159.3	4.9 (0.7, 8.9)	2.2
VO_2 (ml/min)	1753.5±357.3	1590.5±409.8	-9.3	-152.0 (-271.0, 25.4)	0.3
VE (l/min)	35.3 ± 3.0	34.7 ± 3.4	-1.9	-0.7 (-7.0, 5.0)	0.1
BF (breaths/min)	21.7 ± 5.0	21.4 ± 5.0	-1.4	-0.4 (-3.9, 2.8)	0.1
VCO_2 (ml/min)	943.9 ± 87.2	936.6 ± 89.3	-0.7	-29.5 (-156.0, 100.0)	0.1
EqO_2 (l/min)	27.7 ± 1.8	27.6 ± 1.3	-0.4	-0.1 (-2.8, 2.8)	0.1
$EqCO_2$ (l/min)	33.0 ± 3.8	35.4 ± 3.7	7.3	2.1 (-1.3, -6.0)	0.1
HR (beats/min)	95.6 ± 13.4	94.6 ± 13.2	-1.0	-2.8 (-11.8, 7.2)	0.1
VTin (ml)	1.7 ± 0.4	1.7 ± 0.4	-0.1	-0.00 (-0.17, 0.16)	0.1
VTex (ml)	1.7 ± 0.5	1.7 ± 0.5	-0.9	-0.02 (-0.18, 0.14)	0.1
Tin (s)	1.4 ± 0.6	1.4 ± 0.6	1.8	0.02 (-0.21, 0.27)	0.1
Tex (s)	1.7 ± 0.4	1.8 ± 0.4	0.2	0.01 (-0.18, 0.21)	0.1
TiTot (%)	43.2 ± 4.4	40.7 ± 8.9	-5.8	-1.76 (-5.60, 2.14)	0.2
$PETO_2$ (mmHg)	14.8 ± 0.4	14.8 ± 0.4	-0.2	-0.03 (-0.34, 0.30)	0.1
$PETCO_2$ (mmHg)	4.2 ± 0.4	4.2 ± 0.4	-0.3	-0.02 (-0.26, 0.22)	0.1
RER	0.81 ± 0.08	0.80 ± 0.09	-1.2	-0.01 (-0.07, 0.05)	0.1

BF_{10}, Bayes factor; BF, breathing frequency; EqO2, ventilatory equivalent of O_2; $EqCO_2$, ventilatory equivalent of CO_2; HDI, highest density interval; $PETCO_2$, end-tidal CO_2; $PETO_2$, end-tidal O_2; Pre, pre-training test; $Post_{PRE}$, value obtained in the post-training test at the time when the maximum value in the pre-training test was obtained; $Post_{FINAL}$, maximum value obtained in the post-training test; RER, respiratory exchange rate; Tex, expiratory time; Tin, inspiratory time; TiTot, breathing cycle; T_t, total test time; VCO_2, carbon dioxide production; VE, minute ventilation; VE/VCO_2 intercept, intercept of the regression line VE/VCO_2; VE/VCO_2 nadir, lowest point in the curve that describes the relationship VE/VCO_2; VE/VCO_2 slope, slope of the regression line VE/VCO_2; Vtex, expiratory tidal volume; VO_2, oxygen consumption; Vtin, inspiratory tidal volume. 95% highest density intervals that does not include 0 are highlighted in **bold.**
